# *De novo* assembly of the chimpanzee transcriptome from NextGen mRNA sequences

**DOI:** 10.1186/s13742-015-0061-x

**Published:** 2015-04-18

**Authors:** Mnirnal D Maudhoo, Jacob D Madison, Robert B Norgren

**Affiliations:** Department of Genetics, Cell Biology and Anatomy, University of Nebraska Medical Center, Omaha, Nebraska 68198 USA

**Keywords:** *Pan troglodytes*, Chimpanzee, Transcriptome, mRNA-seq, Assembly

## Abstract

**Background:**

Common chimpanzees (*Pan troglodytes*) and bonobos (*Pan paniscus*) are the species most closely related to humans. For this reason, it is especially important to have complete and accurate chimpanzee nucleotide and protein sequences to understand how humans evolved their unique capabilities. We provide transcriptome data from four untransformed cell types derived from the reference *Pan troglodytes*, “Clint”, to better annotate the chimpanzee genome and provide empirical validation for proposed gene models of this important species.

**Findings:**

RNA was extracted from primary cells cultured from four tissues: skin, adipose stroma, vascular smooth muscle and skeletal muscle. These four RNA samples were sequenced on the Illumina HiSeq 2000 platform. Sequences were deposited in the National Center for Biotechnology Information (NCBI) Sequence Read Archive (SRA). Transcripts were assembled, annotated and deposited in the NCBI Transcriptome Shotgun Assembly (TSA) database.

**Conclusions:**

We have provided a high quality annotation of 44,275 transcripts with full-length coding sequence (CDS). This set represented a total of 10,110 unique genes, thus providing empirical support for their existence. This dataset can be used to improve the annotation of the *Pan troglodytes* genome.

**Electronic supplementary material:**

The online version of this article (doi:10.1186/s13742-015-0061-x) contains supplementary material, which is available to authorized users.

## Data description

### Background

Common chimpanzees (*Pan troglodytes*) and bonobos (*Pan paniscus*) last shared a common ancestor with humans approximately six million years ago and are our closest living relatives [1-3]. For this reason, comparisons of DNA, RNA and protein sequences from chimpanzees and bonobos with those of humans are especially useful in understanding the changes that led to the evolution of humans [1-3]. Although they are not often used, biomedical research using common chimpanzees has led to breakthroughs in the study of hepatitis C, and they are the only animal model available to test vaccines for this virus [4]. HIV-1 is derived from a virus found in chimpanzees [5]. Research into how this virus has influenced the evolution of chimpanzees may have important implications for human health [5].

The draft *Pan troglodytes* genome is made up of an initial version of the common chimpanzee genome [1] derived from an animal called “Clint”, plus several updates. However, like all draft genomes, it is incomplete and many of the proposed gene models lack experimental validation. The chimpanzee transcriptome has been investigated using next-generation sequencing (NGS) [6]. Short (35 and 50 bp) reads were aligned against the draft chimpanzee genome using sequencing by oligonucleotide ligation and detection (SOLiD) technology [6]. *De novo* assembly of chimpanzee transcripts has not been attempted.

In the current work, we present 31.2 Gb of Illumina RNA sequencing data of the chimpanzee transcriptome from four different untransformed cell lines derived from “Clint”. These RNA sequences were assembled into 118,995 *de novo* transcripts (deposited at the National Center for Biotechnology Information; NCBI). Of these, 44,275 transcripts contained full-length coding sequences, representing 10,110 unique genes. These datasets of assembled and annotated *Pan troglodytes* transcripts can be used to provide empirical support for conceptually derived gene models for the common chimpanzee genome. Our assembled transcripts can also be used to improve incomplete or incorrect common chimpanzee genome annotations.

### Samples

Four untransformed cell lines, derived from “Clint”, were obtained from the Coriell Institute for Medical Research (Camden, NJ) as follows: fibroblasts from skin (S006007), stem cells from adipose stroma (S008396), endothelial cells from vascular smooth muscle (S008397), and myoblasts from skeletal muscle (S008395).

### Culture and RNA extraction

Cultures of each cell type were grown to near confluence under the conditions recommended by Coriell. Cells were lysed in TRIzol™ (guanidinium thiocyanate and phenol; Ambion/Invitrogen, Carlsbad, Ca.) and RNA was isolated from the aqueous phase using PureLink™ nuclei acid purification system (Ambion/Invitrogen). An RNA Integrity Number (RIN) was determined for each sample using an Agilent Bioanalyzer 2100 (Santa Clara, Ca.). Samples with RINs of ≥9 were utilized for subsequent RNAseq analysis.

### Sequencing

Libraries for all four samples were produced using the Illumina TruSeq RNA Sample Preparation Kit. Sequencing was performed on the Illumina HiSeq 2000 platform (paired-end, 101 base pair reads). A total of 31.2 billion bases of sequence data were deposited to the Sequence Read Archive (SRA; Table 1).

**Table 1:** **Sequence and assembled transcript accessions**

Cells	Tissue type	SRA Accessions	TSA Accessions
Fibroblasts	Skin	SRX179267	GABD01000001–GABD01029686
Stem cells	Adipose stroma	SRX179264	GABC01000001–GABC01029054
Endothelial cells	Vascular smooth muscle	SRX179266	GABF01000001–GABF01029310
Myoblasts	Skeletal muscle	SRX179271	GABE01000001–GABE01030945

### Transcriptome assembly

Illumina reads are often filtered before being used for assembly. We have previously reported that filtering Illumina mRNA sequences for genomic contamination provided high quality transcripts that were useful for annotating a new rhesus genome [7]. A similar strategy was adopted for the chimpanzee. Briefly, we aligned reads with the human Reference Sequencing (RefSeq) mRNA sequences using BLASTn (BLAST+ v2.2.25) [8]. Reads with a reported alignment length <100 and >102 nucleotides were removed from the input file. We also removed a read if its mate was removed.

We used Velvet (v1.2.07) [9] and Oases (0.2.08) [10] to assemble filtered reads into transcripts. The k-mer value was set to 31 for velveth, and the coverage cut-off and expected coverage was set to ‘auto’ in velvetg. Assembled contigs were passed from velvetg to Oases, a transcriptome assembler [10]. Default parameters were used for Oases.

N50 and mean lengths of the contigs associated with each sample were calculated (Table 2). The ranges of values obtained, 2,969-3,349 for N50s and 1,843-2,077 for mean lengths, indicate high quality samples, sequences and assemblies.

**Table 2:** **Submitted contig statistics**

Tissue Type	Number of contigs	N50 length	Mean length	Number of unique full-length CDS
Skin	29,686	2,969	1,896	8,018
Adipose stroma	29,054	2,975	1,907	8,095
Vascular smooth muscle	29,310	2,905	1,843	7,877
Skeletal muscle	30,945	3,349	2,077	8,216

### Annotation

Assembled chimpanzee transcripts were used to query BLASTx [11] (BLAST+ v2.2.25) to identify orthologous human RefSeq proteins. Coding sequence (CDS) ranges obtained from BLASTx output files were used to derive putative chimpanzee protein sequences. Only transcripts with both start and stop codons were annotated. In addition, only transcripts with conceptually derived proteins with protein length differences of ≤10 and protein identities of ≥85% with respect to their human orthologs were annotated in this automated fashion. Both annotated and unannotated transcripts were deposited in the Transcriptome Shotgun Assembly (TSA) database (Table 1).

A total of 118,995 transcripts were deposited to NCBI under BioProject accession number PRJNA173089. From this group, we annotated 44,275 transcripts with full-length CDS, representing a total of 11,438 unique proteins and 10,110 unique genes.

### Comparison with Ensembl annotations

We compared the quantity and quality of our annotated transcripts with Ensembl annotations (Ensembl Genes 78) of the draft chimpanzee genome (CHIMP 2.1.4). Ensembl BioMart [12] was used to obtain Ensembl common chimpanzee protein models and annotations. From the DATABASE menu, we chose Ensembl Genes 78 and DATASET *Pan troglodytes* genes (CHIMP 2.1.4). From Attributes we chose Ensembl Gene ID, Ensembl Transcript ID, Associated Gene Name (equivalent to NCBI’s ‘Gene Symbol’) and Description (equivalent to NCBI’s ‘Gene Description’). Peptide sequences were also obtained from this dataset.

BLASTp was used to align both our assembled transcripts and the Ensembl annotated genome annotations against the human RefSeq proteins [13]. Default settings were used, with the exception of max_target_seqs, which was set to 1. The percent identity, percent similarity and number of gaps were obtained for both assembled transcript (Additional file 1) and Ensembl annotated genome annotations (Additional file 2). Mean values are reported in Table 3. The much lower percent identity and percent similarity values, and higher number of gaps obtained for the Ensembl genome annotations, imply a large number of mistakes in these annotations. We hypothesized this might be due to the inclusion of gene models with no information in the Gene Symbol field. To test this hypothesis, we filtered out Ensembl genome annotations without Gene Symbols. This reduced the number of proteins identified in the Ensembl genome annotation dataset from 19,707 to 18,120 but improved the mean values for percent identity, percent similarity and number of gaps (Table 3). We identified an annotated, assembled transcript from our dataset for 454 unique Ensembl genes with no Associated Gene Name (Additional file 3). In addition, there were 401 unique genes represented in our dataset for which there was no Ensembl protein match with a human ortholog in a BLASTp alignment (Additional file 4).

**Table 3:** **Comparison with Ensembl – all transcripts**

Sequence source	Mean % identity	Mean % similarity	Mean gaps
All assembled transcripts	99.01	99.32	0.24
All Ensembl genome annotations	95.97	96.93	3.45
Filtered Ensemble genome annotations	97.71	98.23	3.24

To provide a direct comparison of our assembled transcripts with Ensembl genome annotations, we first obtained a list of human genes with a single isoform [7], and then found proteins that were contained within this list that had the same Gene Symbols in both our assembled transcript and the Ensembl genome annotations. This selection resulted in a list of 3,552 genes (Additional file 5). Mean values of protein identity, protein similarity and number of gaps were calculated for this common set of genes (Table 4). In this comparison, the difference in quality between assembled transcripts and Ensembl genome annotations is reduced but still noticeable, especially with respect to the mean number of gaps.

**Table 4:** **Comparison with Ensembl – Overlap, single isoform**

Sequence source	Mean % identity	Mean % similarity	Mean gaps
Assembled transcripts	99.43	99.64	0.17
Ensembl genome annotations	98.89	99.13	2.13

## Availability of supporting data

Datasets supporting this article are available at NCBI with BioProject ID: PRJNA173089. Illumina sequences were submitted to SRA and assembled transcripts were deposited to TSA. Accessions for SRA and accession ranges for TSA can be found in Table 1. Supporting data and ISA-TAB metadata are also available from the GigaScience database [14].

## Additional files

Additional file 1:
**BLASTp results for alignment of assembled chimp transcript proteins with human proteins.**
Additional file 2:
**BLASTp results for alignment of Ensembl chimp proteins with human proteins.**
Additional file 3:
**Annotations from assembled transcripts dataset for Ensembl genes with no Associated Gene Name.**
Additional file 4:
**Genes in the assembled transcript dataset for which there is no Ensembl protein.**
Additional file 5:
**BLASTp results for comparison of assembled transcript proteins with Ensembl proteins.**

## Abbreviations

BLASTn: Nucleotide basic local alignment search tool
BLASTp: Protein basic local alignment search tool
BLASTx: Translated basic local alignment search tool
CDS: Coding sequence
RefSeq: Reference sequences
SOLiD: Sequencing by oligonucleotide ligation and detection
SRA: Sequence read archive
TSA: Transcriptome shotgun assembly

## References

[1] Chimpanzee Sequencing and Analysis Consortium (2005). Initial sequence of the chimpanzee genome and comparison with the human genome. Nature.

[2] Wall JD (2013). Great ape genomics. ILAR J.

[3] Ebersberger I, Metzler D, Schwarz C, Pääbo S (2002). Genomewide comparison of DNA sequences between humans and chimpanzees. Am J Hum Genet.

[4] Bukh J (2004). A critical role for the chimpanzee model in the study of hepatitis C. Hepatology.

[5] de Groot NG, Bontrop RE (2013). The HIV-1 pandemic: does the selective sweep in chimpanzees mirror humankind's future?. Retrovirology.

[6] Wetterbom A, Ameur A, Feuk L, Gyllensten U, Cavelier L (2010). Identification of novel exons and transcribed regions by chimpanzee transcriptome sequencing. Genome Biol.

[7] Zimin AV, Cornish AS, Maudhoo MD, Gibbs RM, Zhang X, Pandey S (2014). A new rhesus macaque assembly and annotation for next-generation sequencing analyses. Biol Direct.

[8] Altschul SF, Gish W, Miller W, Myers EW, Lipman DJ (1990). Basic local alignment search tool. J Mol Biol.

[9] Zerbino DR, Birney E (2008). Velvet. algorithms for de novo short read assembly using de Bruijn graphs. Genome Res.

[10] Schulz MH, Zerbino DR, Vingron M, Birney E (2012). Oases. Robust de novo RNA-seq assembly across the dynamic range of expression levels. Bioinformatics.

[11] Gish W, States DJ (1993). Identification of protein coding regions by database similarity search. Nat Genet.

[12] Biomart – Ensembl. 2015. http://www.ensembl.org/biomart/

[13] National Center for Biotechnology Information. ftp://ftp.ncbi.nlm.nih.gov/refseq/H_sapiens/mRNA_Prot/ (2015) Accessed 9 February 2015.

[14] Maudhoo MD, Madison JD, Norgren RB. Supporting data and materials for "De Novo assembly of the chimpanzee transcriptome from NextGen mRNA sequences". GigaScience Database. 2015 http://dx.doi.org/10.5524/100137 10.1186/s13742-015-0061-x PMC4403674 25897398

